# Gait Analysis in Rats with Single Joint Inflammation: Influence of Experimental Factors

**DOI:** 10.1371/journal.pone.0046129

**Published:** 2012-10-05

**Authors:** Kristina Ängeby Möller, Susanne Kinert, Rolf Størkson, Odd-Geir Berge

**Affiliations:** 1 AstraZeneca R&D Södertälje, CNSP iMed Science, Södertälje, Sweden; 2 AstraZeneca R&D Södertälje, Regulatory Affairs, Södertälje, Sweden; 3 Department of Immunology and Transfusion Medicine, Haukeland University Hospital, Bergen, Norway; 4 Multidisciplinary Pain Center, Uppsala University Hospital, Uppsala, Sweden; University of North Dakota, United States of America

## Abstract

Disability and movement-related pain are major symptoms of joint disease, motivating the development of methods to quantify motor behaviour in rodent joint pain models. We used observational scoring and automated methods to compare weight bearing during locomotion and during standing after single joint inflammation induced by Freund's complete adjuvant (0.12–8.0 mg/mL) or carrageenan (0.47–30 mg/mL). Automated gait analysis was based on video capture of prints generated by light projected into the long edge of the floor of a walkway, producing an illuminated image of the contact area of each paw with light intensity reflecting the contact pressure. Weight bearing was calculated as an area-integrated paw pressure, that is, the light intensity of all pixels activated during the contact phase of a paw placement. Automated static weight bearing was measured with the Incapacitance tester. Pharmacological sensitivity of weight-bearing during locomotion was tested in carrageenan-induced monoarthritis by administration of the commonly used analgesics diclofenac, ibuprofen, and naproxen, as well as oxycodone and paracetamol. Observational scoring and automated quantification yielded similar results. We found that the window between control rats and monoarthritic rats was greater during locomotion. The response was more pronounced for inflammation in the ankle as compared to the knee, suggesting a methodological advantage of using this injection site. The effects of both Freund's complete adjuvant and carrageenan were concentration related, but Freund's incomplete adjuvant was found to be as effective as lower, commonly used concentrations of the complete adjuvant. The results show that gait analysis can be an effective method to quantify behavioural effects of single joint inflammation in the rat, sensitive to analgesic treatment.

## Introduction

Joint disease, including rheumatoid arthritis (RA) and osteoarthritis (OA), is an increasing source of sick leave and suffering, partially due to an aging population. In these conditions, disability and movement related pain are major complaints with significant impact on quality of life [1]–[4]. Therefore, disability measures and pain on walking has been used as endpoints to assess effects of different treatments in clinical trials [5]–[10].

Animal models mimicking the clinical situation with regard to tissues and readouts, in this case movement related pain originating from the joint, may facilitate investigation of disease mechanisms as well as development of new symptomatic treatments. Changes in gait and paw pressure of standing and walking rats have been suggested to reflect pain evoked by movement or unwillingness and inability to move the limb after induction of joint inflammation [11]. Commercial equipment has facilitated quantification of inflammation-induced changes in weight load of immobile rodents [12]–[17] and methods for quantification of behaviours interpreted as signs of pain related to movement have also been reported [18]–[21], but are not yet widely implemented. Automated gait analysis equipment is now available and we have reported that an earlier implementation of the CatWalk [22] can be used to quantify individual paw usage parameters as well as parameters related to gait regularity in monoarthritic rats [23]. We showed that paw print area and paw pressure (weight load) are significantly affected by inflammation induced by injection of carrageenan into an ankle joint, presumably due to a combination of pain and motor impairment. These parameters were sensitive to analgesic treatment by morphine and the cyclooxygenase 2 (COX-2) inhibitor rofecoxib.

The aim of the present study was to further investigate the utility of gait analysis for quantification of behaviour induced by joint inflammation and presumably correlated with pain [11]. We wanted to provide a better basis for selection of experimental parameters by characterising the effects of several concentrations of the two commonly used inflammatory agents Freund's complete adjuvant (FCA) and carrageenan and to investigate the impact of injection site, i.e. ankle vs. knee. To facilitate comparison with data obtained with conventional techniques we also visually scored weight bearing while standing and during locomotion after FCA injection and included an experiment with the Incapacitance tester.

The results support the use of gait analysis for quantification of behavioural changes induced by single joint inflammation and indicate that the response is more pronounced during locomotion than when the rats are immobile and when the ankle is affected as compared to the knee. Weight-bearing during locomotion after carrageenan-induced arthritis in the ankle was highly sensitive to analgesics commonly used for joint pain, i.e. the non-steroidal anti-inflammatory drugs diclofenac, ibuprofen and naproxen and the opiate oxycodone and was also affected by higher doses of paracetamol.

## Materials and Methods

### Ethics statement

These studies were approved by the Stockholm Södra Animal Research Ethical Board, with approval numbers S14/02, S 19/04, S 120/06 and S 111/07.

### Animals and housing

In total, 467 Sprague-Dawley male rats (Scanbur B&K Universal, Sollentuna, Sweden), weighing 200–360 g at the start of testing were used. The animals were housed 3–6 per cage in transparent Macrolon® cages with wood shavings as bedding, with free access to food (R70, Lactamin AB, Vadstena, Sweden) and tap water. The lighting was controlled with 11.5 h daylight, 11.5 h darkness, 0.5 h dusk and dawn. The animals were acclimatized for at least one week before being subjected to experimental procedures and were habituated in the test room for at least 30 minutes before testing. Treatments were randomized by a computer program and the observer was blinded to group assignment when possible. However, the swelling of joints injected with FCA or carrageenan was in many cases obvious and all animals in the study using visual scoring received the same treatment and were recorded on the same days.

### Apparatus

To measure weight bearing and gait regularity during locomotion, the animals were allowed to traverse a walkway (black acrylic walls, placed 10 cm apart; length 100 cm, height 21 cm) with a 0.6 cm thick glass floor as previously described [22], [23]. The walkway has an entrance at one end and an exit with a sliding door towards the goal-cage. Light is projected via 100 fibre optic cables connected to a 150 W light source into the long edge of the glass floor. There is virtually complete reflection of the light within the floor except where an object, such as a rat paw, touches the glass causing light to be scattered, producing an illuminated image [24]. The light intensity of the image depends on the degree of contact against the floor and reflects the pressure exerted at that point [24], [25]. Low intensity green background light provides an image of the animal separable from the white paw prints and is used to calculate position and direction of the animal. Images are captured by a video camera positioned 1 m below the floor and stored on video for optional reanalysis and quality check.

For visual observations during restricted movements, the animals were placed individually in an acrylic observation chamber (length 20 cm, width 10 cm and height 18 cm) on a glass floor, as previously described [12]. A video camera was mounted directly below the cage at a distance of 1 m and recordings were stored for later analysis.

Weight bearing was also measured on the Incapacitance tester (Linton Instruments) using modified restrainers. The animals were placed in the restrainers with their hind paws on separate sensors registering the weight of each hind paw.

### Automated gait analysis

**Figure 1:**
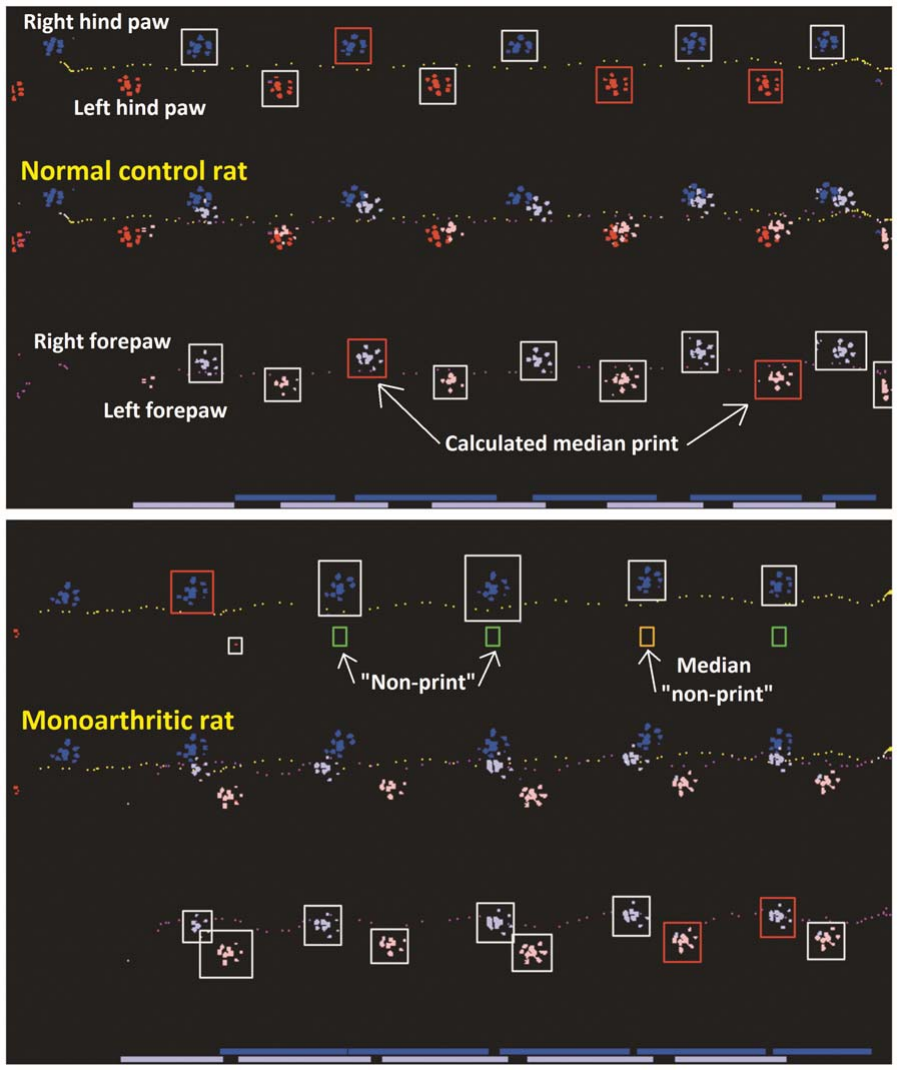
Prints from rats in the PawPrint walkway. The walking pattern of a normal control rat (upper panel) and a representative monoarthritic rat (lower panel). The inflammatory induction agent was injected into the ankle joint of the left hind paw. The middle trace of each panel shows prints from the left side in red, from the right side in blue. Prints from forepaws are shown in light colour, also seen separately in the lower trace, whereas prints from the hind paws are darker, and shown separately in the upper trace. Each print detected by the PawPrint algorithm is surrounded by a white rectangle, but coloured red for the calculated median print. In places where prints would have been expected in normal gait, but not actually detected, green rectangles occur. When such a “non-print” is chosen as the median print, the rectangle is coloured orange.

A custom made computer program, the PawPrint, was used. Data acquisition and analysis of weight bearing and gait regularity based on video capture (25 frames/sec) from the walkway starts automatically when the algorithm identifies the paws, and continues as long as the rat keeps moving forward. Rearing >1.5 seconds, small movements in the opposite direction for more than 160 ms, any large movement in the opposite direction, or the program loosing track of the animal, stops the analysis. Uninterrupted locomotion to allow registration of at least 3 paw placements by each non-arthritic paw is required. No satisfactory recording within four walkway crossing attempts will generate a missing value, but this did not occur in the studies presented here. After a successful crossing the program immediately calculates a number of parameters pertaining to the rat's gait pattern and weight bearing. Data and a picture of the prints in false colours for easy identification of each paw are displayed (Fig. 1), allowing the experimenter to make a quality check of the results. The choice of parameters was based on results from the CatWalk [23].

#### Weight bearing per paw

Pixels showing intensities above a threshold value of 50 (range 0–255, arbitrary unit) are defined as contact points and are automatically assigned to the relevant paw. For the duration of each paw placement, the maximum value of light intensity (*I*) in arbitrary units is recorded for each pixel assigned to that paw. The sum of these values is an area-integrated paw pressure, i.e. the dynamic (vertical) weight bearing of the particular paw placement. For every print from a paw, the weight bearing during locomotion is measured according to the formula:
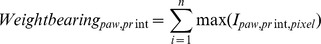
For each paw, the median value of all paw placements captured in a passage is calculated, providing one value in arbitrary units when the passage is completed. In order to estimate each paw's relative contribution, the value is recalculated in per cent of the sum of values for all paws.

#### Guarding index

Guarding index is calculated as the difference in percent dynamic weight bearing between the two hind paws, in order to capture any shift of weight between the hind paws due to arthritis.

#### Regularity index

A normal step sequence is made up of all four paws placed one after the other and the degree of interlimb coordination can be expressed as the regularity index, calculated as follows:
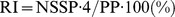
where NSSP represents the number of normal step sequence patterns and PP the total number of paw placements [22].

### Induction of monoarthritis

Under deep anaesthesia (5% isoflurane in oxygen/breathing air), 50 µL of either induction agent was injected with a 21-gauge needle into the left tibio-tarsal joint from the dorsal side, or into the left knee joint. FCA (Freund's complete adjuvant; Sigma-Aldrich containing 1.0 mg heat killed and dried Mycobacterium tuberculosis per mL) was used except for the concentration response study, where FCA was prepared by adding 100 mg of Mycobacterium tuberculosis (Difco laboratories) to 12.5 mL of Freund's incomplete adjuvant which was then diluted to complete the concentration range (0.12, 0.25, 0.5, 1.0, 2.0, 4.0 and 8.0 mg/mL). Carrageenan (Sigma-Aldrich, Sigma Chemical CO St Louis, MO, USA) was dissolved in physiological saline. The injection was completed in less than one minute, and rats recovered from the anaesthesia within two to three minutes. To ensure that the injection was truly intra-articular, control experiments were performed in advance using Evan's blue. It cannot be excluded that a small portion of the injected volume leaked outside the articular capsule although no evidence of this was observed.

### Visual scoring of weight bearing

**Figure 2:**
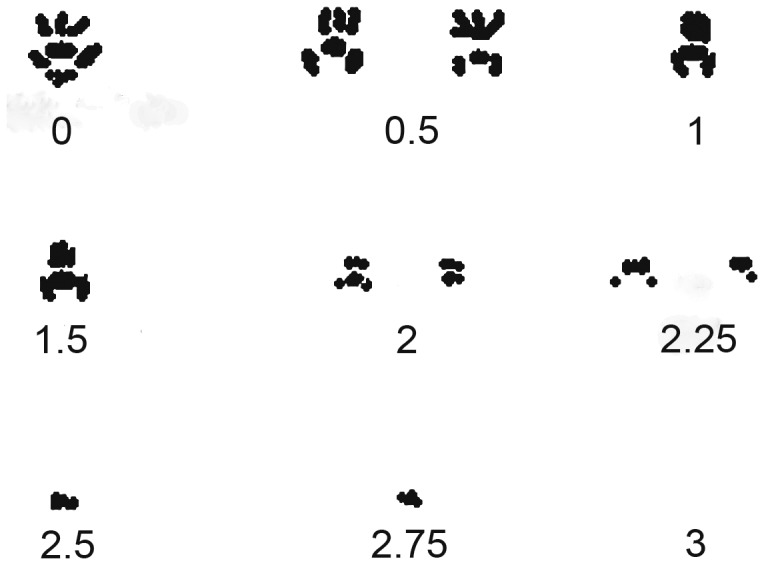
Visual rating scale for weight bearing during locomotion. Illustration of typical paw prints reflecting the rating scale used. In addition to the visual print pattern, the following criteria were implemented: 0 was assigned to rats where no difference could be detected between the two hind paws, 1.5 required the animals to limp, 2.5 was assigned when each step cycle was complete, i.e. that the affected paw touched the floor at every step, 2.75 when only some of the cycles were complete, and 3 when the paw remained off the floor for the entire crossing.

Monoarthritis of the ankle joint was induced in 24 rats by injection of FCA (1.0 mg/mL). Testing took place 2, 6 and 8 days later in order to obtain a distribution of scores. On each occasion, the animals were first recorded in the walkway and then in the observation chamber described above. An extended form of the rating scale previously described by Coderre and Wall [11] was used (Table 1). A similar scale was developed for the walkway (Fig. 2). The same observer analyzed all data to avoid inter-observer variation.

**Table 1:** Paw pressure visual rating scale in rats filmed in observation chambers.

Score	Criteria
**0**	Normal paw pressure, equal weight on both hind paws
**0.5**	Normal paw pressure, paw is completely on the floor but toes are unequal to control hind paw
**1**	Slightly reduced paw pressure, paw is completely on the floor but toes are not spread
**1.5**	Reduced paw pressure, intermediate between category 1 and 2
**2**	Moderately reduced paw pressure, paw curled with only some parts of the hind paw lightly touching the floor
**2.5**	Moderately reduced paw pressure, paw curled with toes only lightly and occasionally touching the floor
**3**	Severely reduced paw pressure, paw completely elevated

### Automatic assessment of weight bearing during locomotion

Handling and testing was performed during the light phase in a room with lights dimmed. The animals were allowed to habituate in and to pass through the walkway once daily on three or four occasions before monoarthritis induction. Before each training or test session all animals from a home cage were transferred to the goal-cage at the exit of the walkway. In the first training session, goal-cage habituation lasted for about five minutes. On the following occasions the habituation lasted 2–3 minutes, whereas on the day of the experiment the first animal was brought to the walkway entrance after a maximum of one minute in the goal-cage. Training and testing were conducted by the same person.

#### FCA: concentration- and time response

In a second experiment, Freund's complete adjuvant (Difco laboratories) was injected into the ankle joint in concentrations of 0.12, 0.25, 0.50, 1.0, 2.0, 4.0 or 8.0 mg/mL and compared with two groups of controls; either naïve rats or rats injected with Freund's incomplete adjuvant (FIA). Recordings were made before as well as 5 hours and between 1 and 21 days after induction of monoarthritis.

#### Carrageenan: concentration- and time response

In a third experiment, naïve rats were compared to animals receiving one of seven concentrations of carrageenan into the ankle joint; 0.47, 0.94, 1.9, 3.8, 7.5, 15 and 30 mg/mL. Recordings were made before and 3, 5, 7, 24 and 48 hours after injection.

#### Comparison between monoarthritis in ankle and knee

Included in the second experiment were separate animals tested before and 5 hours as well as 1–21 days after intra-articular injection of FCA (1 mg/mL; Sigma Aldrich) in the ankle or knee. In a fourth experiment, time-points for testing after carrageenan (7.5 mg/mL) were before as well as 3 and 5 hours and 1, 2, 3, and 4 days after induction (n = 10 per experimental group; 5 control animals were injected into the ankle and 5 into the knee with saline).

### Assessment of static weight bearing using the Incapacitance tester

In a fifth experiment, animals were habituated to the test equipment for 5 minutes once daily during three days before the day of experiment. Static weight bearing was measured on the Incapacitance tester (Linton Instruments). The animals were placed in the restrainers with their hind paws on separate sensors registering the weight of each hind paw, and allowed to settle for about one minute before 5 recordings, each lasting 3 seconds, were made. The recordings were completed within 4 minutes. Data for each hind paw was expressed in percent of the hind paws' total static weight bearing.

Naïve rats and rats injected with carrageenan (7.5 mg/mL) into the ankle or knee were tested in a cross-over design where 5 rats from each treatment group were tested on the walkway first and then on the Incapacitance tester, whereas the other 5 rats from each treatment group were tested in the reverse order. Recordings were made before and 3, 5 and 24 hours after induction of monoarthritis.

### Pharmacological testing and bioanalysis

In additional pharmacological experiments the following drugs were tested for antinociceptive effects in the carrageenan induced monoarthritis: naproxen, diclofenac, oxycodone (Sigma Aldrich), paracetamol (Fluka) and ibuprofen (synthesized at AstraZeneca). Vehicle or drugs were administered 1 hour before (ibuprofen, diclofenac, and paracetamol) or 1 hour (naproxen) or 2.5 hours (oxycodone) after ankle joint injection of carrageenan. Paracetamol was dissolved in 0.5% methyl cellulose, whereas naproxen, ibuprofen, diclofenac and oxycodone were dissolved in 0.9% physiological saline. Naproxen was given *via* gavage in a volume of 2 mL/kg body weight; ibuprofen, diclofenac and paracetamol *via* gavage in a volume of 5 mL/kg body weight; and oxycodone injected subcutaneously in a volume of 2 mL/kg body weight. Testing was performed 3 hours after carrageenan except for the naproxen experiment when testing took place 5 hours after carrageenan injection.

Plasma samples were taken from the tail vein from 3 satellite animals per treatment group at the time of testing or from all the tested animals at termination of the experiment corresponding to about 5 hours 20 minutes after administration of naproxen, 3 hours after ocycodone and 7 hours after administration of ibuprofen, diclofenac and paracetamol. Analysis of plasma exposure was performed using a bioanalytical method that was developed for each drug for determination of concentrations in the low nanomolar range in plasma samples by reversed-phase liquid chromatography and electrospray tandem mass spectrometry. The pre-treatment of plasma was based on protein precipitation. The quantification of unknown samples was performed using the mass spectrometer software. Standard curves represented by the plots of the peak area ratios of the analytes to internal standard (warfarin) versus concentrations of the standard samples were generated. The concentrations for the unknown samples were calculated from regression equation where quadratic curves with 1/X^2^ weighting or best curve fit were used.

### Data analysis and statistics

Data are presented as mean values ± SEM (n = 8–10 per group unless otherwise stated). The non-parametric Kendall rank correlation coefficient test was used to analyse differences between the two visual rating scales, and the relationship between them is shown with linear regression. The results obtained from the PawPrint algorithm and from the Incapacitance tester were subjected to repeated measures analysis of variance (ANOVA) with subsequent post hoc comparisons using Bonferroni's test. In the pharmacological experiments, 1-way ANOVA followed by Dunnett's Multiple Comparison test was performed to establish which doses showed significant reduction of the guarding index compared to vehicle treated animals. A *p* value of less than 0.05 was considered significant.

## Results

### Comparing weight bearing while standing and during locomotion using visual scoring

**Figure 3:**
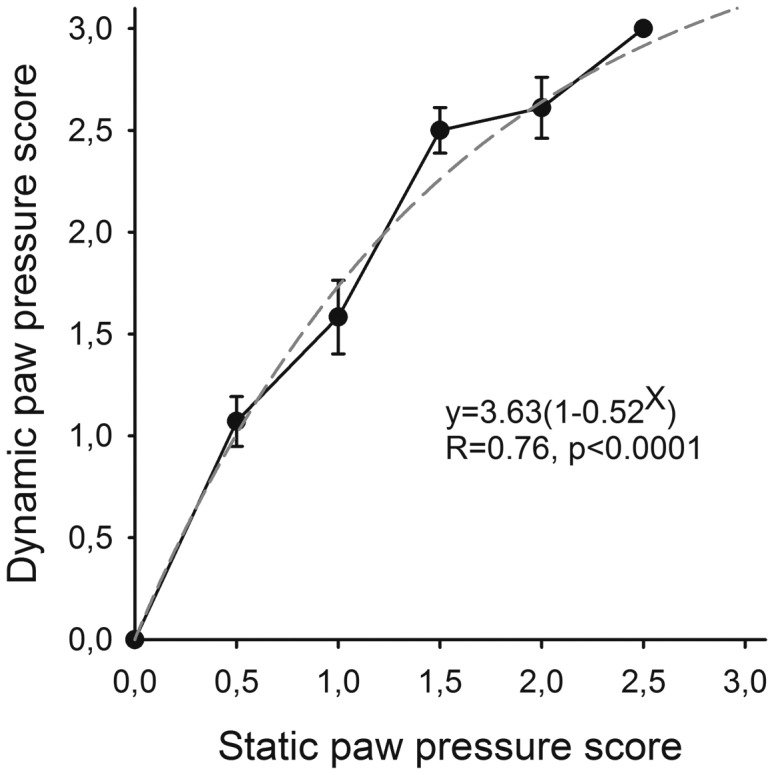
Correlation between ratings of weight bearing while standing and during locomotion after FCA-induced arthritis of the ankle. The visual rating scores on the walkway (during walking) corresponding to each conventional (while standing) observation chamber score are presented as mean ± SEM. The dashed line represents an exponential curve fit based on all paired observations.

The 24 rats with FCA-induced monoarthritis tested both on the walkway and in the observation chambers provided 70 paired observations in total (2 missing values; Fig. 3). Scores during locomotion were higher throughout the rating scales and score 3 was assigned to 9 animals on the walkway but to none in the static situation. Overall, the scores in the two paradigms were highly correlated with an exponential relationship (P<0.001).

### Weight bearing of control animals during locomotion

There were no significant differences in weight bearing between left and right sides of either fore- or hind paws in non-injected naïve or saline-injected control animals and the control groups remained within the same range during the studies, resulting in values for guarding index close to zero (not shown).

### Effects of different concentrations of induction agents on weight bearing during locomotion

#### Freund's complete adjuvant

**Figure 4:**
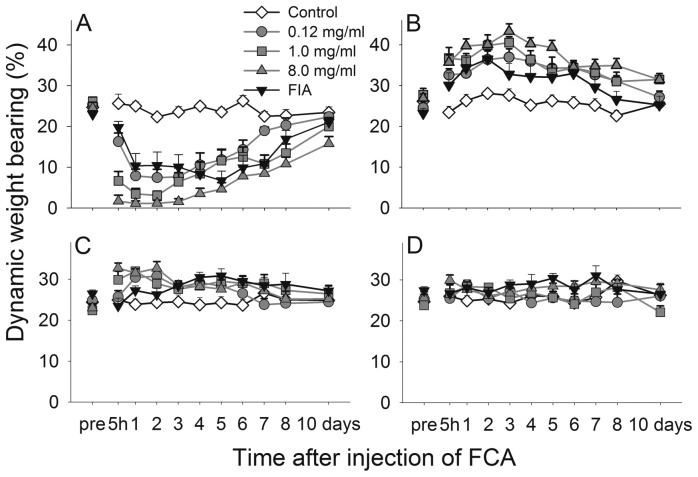
FCA ankle joint injection affects weight bearing of the four paws during locomotion differently. Time course of the weight bearing of the injected left hind paw (A), the non-injected right hind paw (B), the left forepaw (C) and the right forepaw (D), in naïve control rats and rats before and after induction of monoarthritis by injecting FCA or Freund's incomplete adjuvant into the ankle joint. For clarity, data from some concentrations (0.25, 0.50, 2.0 and 4.0 mg/mL) are omitted. Data shown as mean and SEM, n = 8–9 per group.

Ankle joint injection of FCA caused a concentration related reduction in the weight bearing of the injured paw during locomotion (Fig. 4 A; P<0.001 for group, time and interaction effects, 2-way repeated measures ANOVA). Compared to naïve rats, the lowest concentration (0.12 mg/mL) significantly reduced weight bearing from the five hour time point up to six days after injection while the highest concentration (8.0 mg/mL) reduced the scores up to ten days.

The non-injected hind paw was significantly affected in the opposite direction (Fig. 4 B; P<0.001 for group, time and interaction effects, 2-way repeated measures ANOVA), showing an increase in weight bearing for all concentrations of FCA up to eight days after injection. A smaller increase could also be seen in the ipsilateral forepaw, significant one day post injection after all concentrations, and lasting for two days for the high concentration (Fig. 4 C; P = 0.066 for group effect, P<0.001 for time effect and interaction, 2-way repeated measures ANOVA) while the contralateral forepaw was not significantly affected (Fig. 4 D).

Regardless of paw, Freund's incomplete adjuvant caused the same degree of effect as the 0.12 mg/mL concentration of FCA. Between 12 and 21 days after injection no group showed scores significantly different from pre-injection values (data not shown).

**Figure 5:**
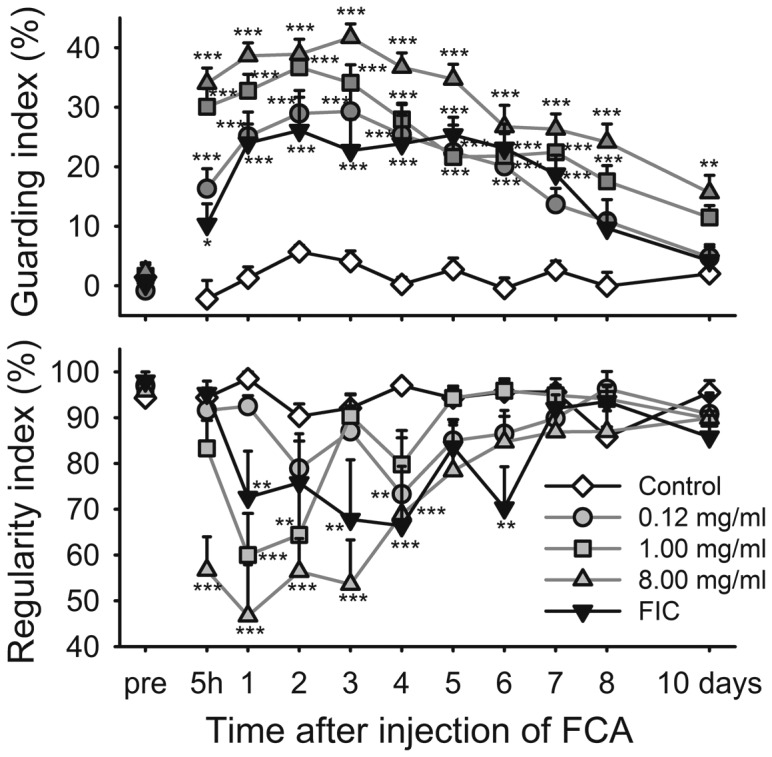
FCA ankle joint injection increases guarding and reduces regularity during locomotion. Time course of guarding index (upper panel) and regularity index (lower panel) in naïve control rats and rats before and after induction of monoarthritis by injecting FCA or Freund's incomplete adjuvant into the ankle joint. Bonferroni's test subsequent to ANOVA: * = *p*<0.05, ** = *p*<0.01, *** = *p*<0.001 compared to the naïve control group at the same time point. Data shown as mean and SEM, n = 8–9 per group.

The guarding index was increased by all concentrations of FCA but also by Freund's incomplete adjuvant (Fig. 5, upper panel). The effect was concentration related but only the higher concentrations of 4.0 and 8.0 mg/mL were significantly more effective than the incomplete adjuvant (not shown). The regularity index (Fig. 5, lower panel) was decreased but significantly so only for four days after the high concentration, with large variability in all groups.

No spreading of the inflammation from the injected joint to other sites was observed over the three week period after FCA injection.

#### Carrageenan

**Figure 6:**
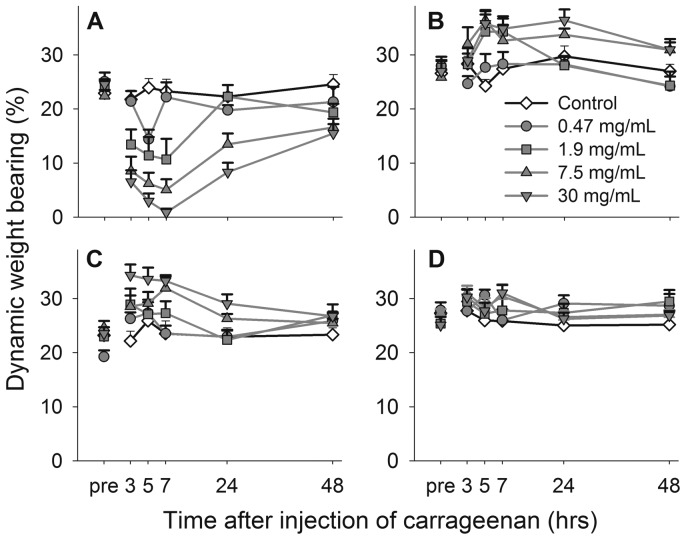
Carrageenan ankle joint injection affects weight bearing of the four paws during locomotion differently. Time course of the weight bearing of the injected left hind paw (A), the non-injected right hind paw (B), the left forepaw (C) and the right forepaw (D), in control rats and rats before and after induction of monoarthritis by carrageenan in the ankle joint. Concentrations omitted for clarity (0.94, 3.8 and 15 mg/mL) follow the same pattern of response, in between the results of those shown. Data shown as mean and SEM, n = 10 per group.

Even injection of carrageenan into the ankle reduced the weight bearing during locomotion of the affected paw in a concentration related manner (Fig. 6 A; P<0.001 for group, time and interaction effects, 2-way repeated measures ANOVA). The maximum effect was observed at 7 hours except for the lowest concentration (0.47 mg/mL) which had a significant effect at the five hour time point only. The effect of the highest concentration (30 mg/mL) was significant at all time points.

The contra-lateral hind paw was also affected (Fig. 6 B; P = 0.006 for group effect, P<0.001 for time effect and P = 0.002 for interaction, 2-way repeated measures ANOVA), and showed a significant increase for 1.9, 7.5 and 30 mg/mL at five hours after injection. An increase was also seen for the ipsilateral forepaw after 7.5 and 30 mg/mL up to seven hours post injection (Fig. 6 C; P<0.001 for group effect, P<0.001 for time effect and P = 0.017 for interaction, 2-way repeated measures ANOVA). The contralateral forepaw did not differ significantly from control animals (Fig. 6 D).

**Figure 7:**
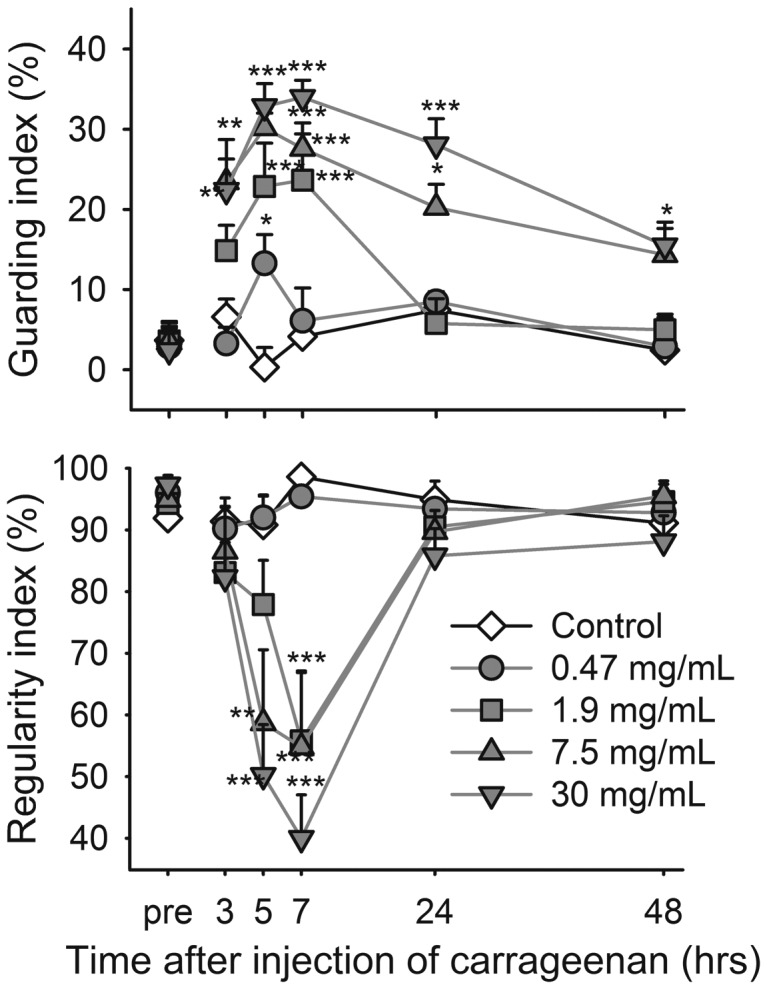
Carrageenan ankle joint injection increases guarding and reduces regularity during locomotion. Time course of guarding index (upper panel) and regularity index (lower panel) in control rats and rats before and after induction of monoarthritis by carrageenan in the ankle joint. Bonferroni's test subsequent to ANOVA: * = *p*<0.05, ** = *p*<0.01, *** = *p*<0.001 compared to the control group at the same time point. Data shown as mean and SEM, n = 10 per group.

Compared to controls, all concentrations increased the guarding index in a concentration-related manner (Fig. 7, upper panel). The regularity index (Fig. 7, lower panel) was significantly decreased at the 5 and 7 hour time points.

### Comparing monoarthritis in ankle joint and knee joint

**Figure 8:**
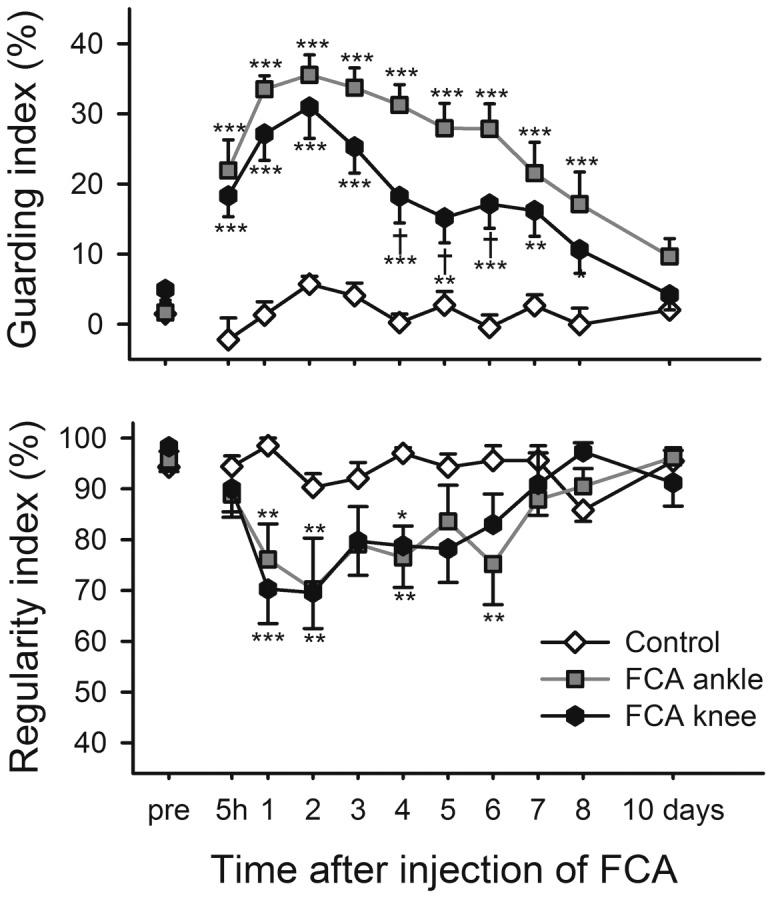
Injection site of FCA plays a role for magnitude of response during locomotion. Time course of the guarding index (upper panel) and regularity index (lower panel) for control rats and rats injected with FCA into the ankle or knee joint. Bonferroni's test subsequent to ANOVA: * = *p*<0.05, ** = *p*<0.01, *** = *p*<0.001 compared to the control group at the same time point and † = *p*<0.05 when comparing ankle to knee injection groups. Data shown as mean and SEM, n = 10 per group.

FCA (1.0 mg/mL) increased the guarding index whether injected into the ankle or the knee (Fig. 8, upper panel; P<0.001 for group, time and interaction, 2-way repeated measures ANOVA). The ankle joint injection yielded values significantly higher than knee joint injection at four to six days. Regularity index decreased to a minimum of 70% one to two days after injection (P = 0.012 for group effect, P<0.001 for time effect and P = 0.030 for interaction, 2-way repeated measures ANOVA) but there was no significant difference at any time between the FCA-injected groups (Fig. 8, lower panel). Both FCA and injection site affected weight gain after injection (not shown). FCA retarded weight gain during the first 4 days but subsequently rats with ankle joint injection gained weight at the same rate as controls, remaining 30–35 g below the control rats at all times points. The animals injected into the knee reached control levels 3 weeks after the injection.

**Figure 9:**
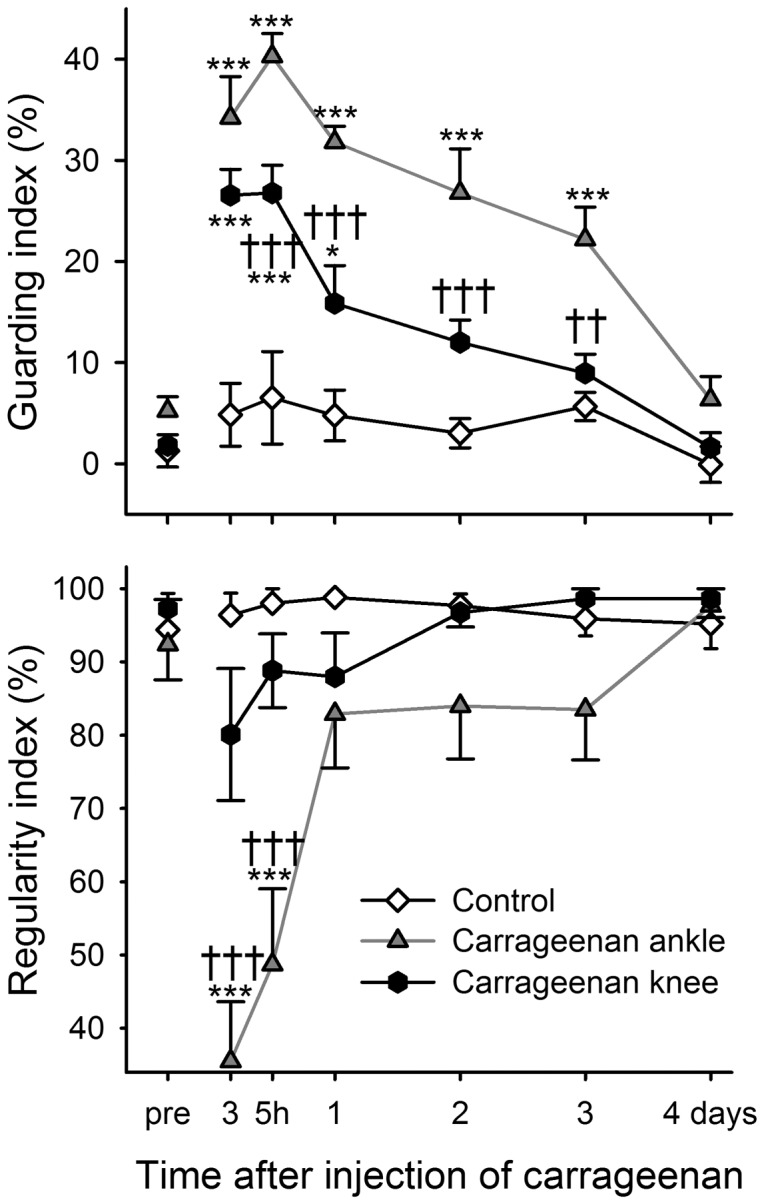
Injection site of carrageenan plays a role for magnitude of response during locomotion. Time course of guarding index (upper panel) and regularity index (lower panel) for control rats and rats injected with carrageenan into the ankle or knee joint. Bonferroni's test subsequent to ANOVA: *** = *p*<0.001 compared to the control group at the same time point and †† = *p*<0.01, ††† = *p*<0.001 when comparing ankle to knee injection groups. Data shown as mean and SEM, n = 10 per group.

The effect of injection site on guarding index appeared greater after carrageenan (7.5 mg/mL) than after FCA, animals with ankle injection showing significantly higher values than knee injected rats between 5 hours and 3 days (Fig. 9, upper panel; P<0.001 for group, time and interaction effects, 2-way repeated measures ANOVA). Also regularity index was significantly affected after ankle joint injection with reductions of mean regularity index to 36% at 3 hours and 49% at 5 hours, whereas knee injection failed to significantly affect this parameter at any time point (Fig. 9, lower panel, P<0.001 for group, time and interaction effects, 2-way repeated measures ANOVA).

### Effect of carrageenan injection site on weight bearing during locomotion vs. standing

**Figure 10:**
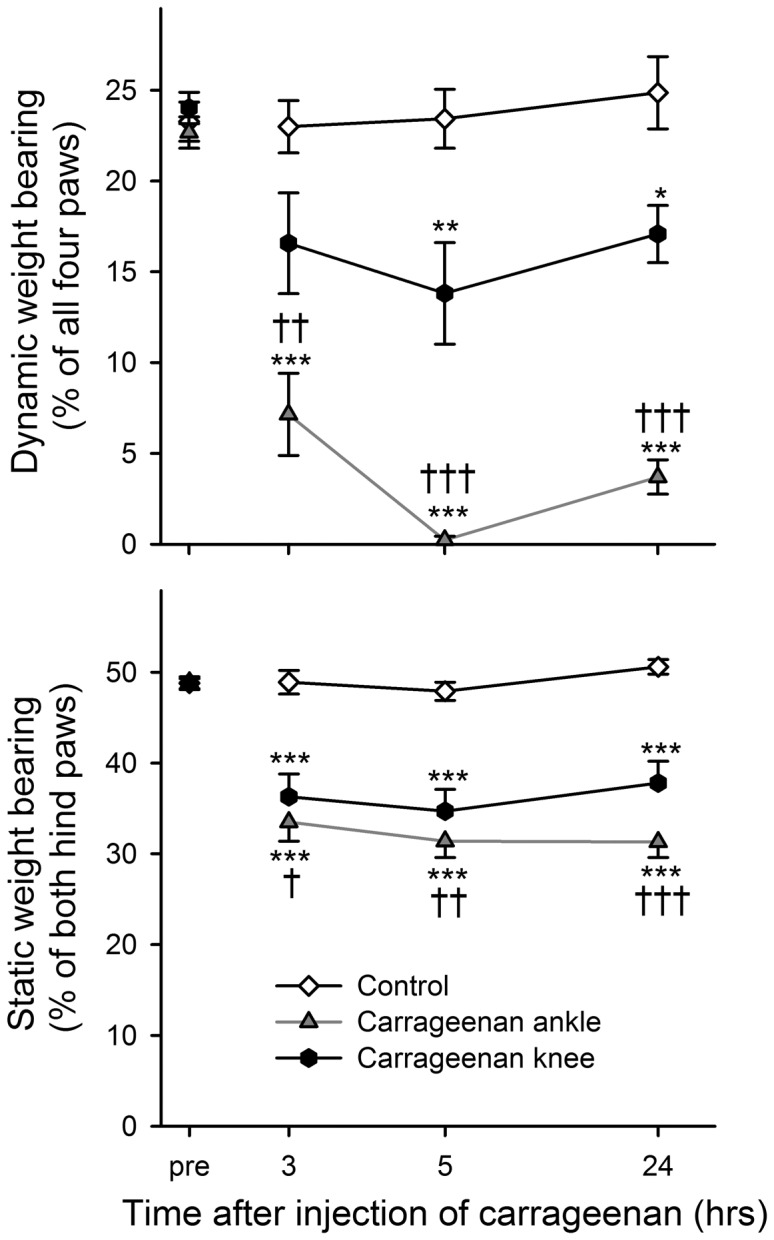
Magnitude of response depends on injection site as well as testing paradigm. Time course of weight bearing for control rats and rats injected with carrageenan into the ankle or knee joint. Upper panel: weight bearing during locomotion in the PawPrint paradigm. Lower panel: Static weight bearing of the same animals in the Incapacitance tester. Bonferroni's test subsequent to ANOVA: * = *p*<0.05, ** = *p*<0.01, *** = *p*<0.001 compared to the control group at the same time point and † = *p*<0.05, †† = *p*<0.01, ††† = *p*<0.001 when comparing ankle to knee injection groups. Data shown as mean and SEM, n = 10 per group.

As in the previous experiment, rats injected with carrageenan (7.5 mg/mL) showed a difference in guarding index between injection sites. The mean weight bearing during locomotion decreased from about 25% to a minimum of 0.2±0.2% after ankle joint injection compared to 13.8±2.8% after injection into the knee joint (Fig. 10 A, P<0.001 for group, time and interaction effects, 2-way repeated measures ANOVA). In the same rats, static weight bearing on the Incapacitance tester was similarly affected with a more pronounced effect after ankle injection but overall, the reduction in weight bearing was smaller in this paradigm (Fig. 10 B, P<0.001 for group, time and interaction effects, 2-way repeated measures ANOVA).

### Effect of analgesics

Single administration of the tested compounds produced dose-related reductions in the response to carrageenan as measured by the guarding index (Table 2). Significant effects were seen at doses of 10 (naproxen), 100 (ibuprofen), 1 (diclofenac), 3 (oxycodone) and 2000 (paracetamol) µmol/kg, corresponding to 2.3 (naproxen), 23 (ibuprofen), 0.30 (diclofenac), 1.1 (oxycodone) and 300 (paracetamol) mg/kg. The doses are not directly comparable due to differences in administration and sampling protocols necessitated by practical considerations. All compounds produced dose-related total plasma exposures.

**Table 2:** Effect of reference compounds on carrageenan induced monoarthritis.

			Total plasma exposure (µM)	
Test compound	Dose (µmol/kg)	Guarding index (mean ± SEM)	At testing	At termination
**Naproxen (N = 8)**	0	29.5±3.5		
	1	24.4±4.1	-	2.5±0.6
	10	10.0±2.9**	-	35±8.5
	100	−4.9±4.9***	-	390±72
**Ibuprofen (N = 8)**	0	26.8±5.9		
	30	14.9±5.6	-	2.8±0.2
	100	7.0±3.7*	-	14±1.7
	300	1.7±3.0**	-	50±6.8
**Diclofenac (N = 8)**	0	27.4±2.5		
	1	14.4±3.7*	0.025±0.008	0.016±0.000
	3	8.9±5.7**	0.068±0.008	0.033±0.004
	10	0.0±3.1***	0.320±0.028	0.130±0.021
**Oxycodone (N = 10)**	0	18.9±4.4		
	1	21.4±4.6	0.088±0.014	<LoQ†
	3	2.1±3.3**	0.360±0.110	<LoQ†
	10	2.5±2.7**	1.9±1.0	0.032±0.022
**Paracetamol (N = 10)**	0	23.4±3.3		
	1000	18.3±3.8	150±550	68±30
	2000	8.1±3.0**	340±120	280±27
	4000	4.7±3.0**	560±100	320±59

† LoQ = limit of quantification.

**Note:** Testing was performed 3 hours after carrageenan injection except for the naproxen experiment when testing took place 5 hours after carrageenan. Oxycodone was given subcutaneously 30 min before test while all other compounds were administered per os 4 hours before testing. Plasma samples were taken from satellite animals (n = 3 per group) at the time of testing or from the tested animals at termination of the experiment corresponding to about 5 hours 20 min after administration of naproxen, 3 hours after oxycodone and 7 hours after ibuprofen, diclofenac and paracetamol. Statistically different from vehicle group:

* = *p*<0.05,

** = *p*<0.01,

*** = *p*<0.001, Dunnett's Multiple Comparison test subsequent to 1-way ANOVA.

## Discussion

These data confirm that single joint inflammation in a hind limb causes quantifiable behavioural changes in weight bearing and gait. Whether the quantification is performed by traditional visual observation or by automated or semi-automated methods, a stronger response is evident during locomotion. We show here that there are more pronounced behavioural responses and more persistent effect on body weight gain when arthritis is induced in the ankle as compared to the knee, suggesting that injection site plays a role for the severity of the insult. Overall, the effect on behaviour is related to the amount of induction agent but surprisingly, Freund's incomplete adjuvant was as effective as lower, commonly used concentrations of FCA. Using carrageenan as an induction agent, we show that gait analysis is sensitive to various classes of analgesics at clinically relevant exposure levels.

We used both visual scoring and two methods of automated or semi automated quantification of behaviour. Visual scoring of video recordings can minimize experimenter-animal interaction but the scoring is limited to a set of arbitrary categories and although proper blinding and randomisation will reduce or eliminate observer bias, extensive calibration is required to obtain consistent results over time and between observers. The PawPrint and commercially available automated gait analysis systems allows testing with a minimum of experimenter-animal interaction and the advantage of consistent, objective scoring and a continuously distributed readout.

The Incapacitance tester also provides a continuous readout but requires the rats to be restrained. Restraint has been found to reduce responses to noxious thermal stimuli [26], [27], and to potentiate responses to opioids in acute thermal nociceptive tests [28], [29]. Peripheral temperature drops by stress, but stress can also have a direct antinociceptive effect in the rat [30]. The walkway paradigms used here allow the animals to freely explore the test apparatus and locomotion occurs without force or prompting, presumably causing a minimum of stress during testing.

We found that categorical, visually obtained guarding scores from immobile rats correlated to scores in the same rats during locomotion, but with higher scores in the latter situation. The PawPrint and Incapacitance tester data revealed that the reduced weight bearing on the affected paw was compensated by a shift of weight to the contralateral hind paw, but the magnitude was smaller on the Incapacitance tester, again indicating more discomfort as the paw is used for movement. Struggle or vocalization in response to flexion/extension of an inflamed joint has been used as a measure of joint hyperalgesia [31]–[34] and found to correlate with paw pressure in the CatWalk [19]. The increased signal during locomotion improves the possibility for pharmacological testing, and is probably relevant for movement-induced pain in joint disease. The data suggest that it is warranted to consider weight bearing during locomotion and during standing as separate measures. However, a compound measure has been suggested by Ferreira-Gomes et al [19].

We have previously shown pharmacological efficacy of morphine and rofecoxib in the CatWalk paradigm [23] and here we extend these findings by demonstrating dose-related efficacy of several non-steroidal anti-inflammatory drugs as well as paracetamol and the opiate oxycodone. It should be pointed out that the studies were not optimized with regard to the pharmacokinetic properties of the compounds so the data should be interpreted with caution. Nevertheless, the potencies were within or close to the clinical single dose range for all compounds except paracetamol. The low potency of the latter compound may in part be explained by a short plasma half-life [35] but plasma levels at the time of testing were at the higher end of the clinically accepted range of 10–20 µg/L even for the ineffective dose. There is evidence that high doses of paracetamol in rodents activate opioid mechanisms [36].

Rodent pain models vary in sensitivity to different classes of analgesics and within a particular model, the sensitivity varies with read-out [37]. The nearly complete reversal of arthritis-induced behaviour observed at clinically relevant doses and exposures in the present study suggest that carrageenan induced arthritis in combination with gait analysis is highly sensitive to both cyclooxygenase inhibition and opiate effects. The efficacies are, however, greater than what would be expected in clinical conditions like osteoarthritis [38], suggesting that relevant pharmacodynamic mechanisms, but not the full pathophysiology, are modelled in the current paradigm. Further pharmacological studies with other induction agents are therefore warranted.

The time course of the effects of the two induction agents is in agreement with previous reports [39]–[42] and with analysis of biochemical biomarker levels in synovial fluid [43]. Different induction agents may recruit different pathophysiological mechanisms and it is therefore interesting that Freund's incomplete adjuvant was as effective as commonly used concentrations of the complete adjuvant indicating that higher doses may be required for specific FCA-mediated effects. Although some authors report that mineral oil does not cause symptoms similar to FCA [32], [44], there are others showing that non-immunogenic adjuvant can induce arthritis in rats [45], [46].

Either induction agent resulted in higher scores when the ankle joint was affected as compared to the knee. The effect was more pronounced during locomotion. In the rat ankle joint, injection volumes greater than 50 µL causes firm resistance [31] while volumes up to 200 µL have been injected into the knee joint [47], [48]. In our studies we injected 50 µL into either joint and the local pressure may have been greater in the ankle. This is probably not a critical factor as both joints swell markedly after injection of either induction agent indicating that inflammation spreads outside the joint capsule regardless of whether the injection fills the joint completely. Pointing in the same direction is data showing development of monoarthritis after injection of FCA into the soft tissue surrounding the ankle joint, demonstrating an initial inflammatory response followed by histological evidence of chronic monoarthritis [49]–[51]. During locomotion, rats with inflammation of the knee seemed to avoid bending of the injured joint by placing the paw further to the side, whereas animals with ankle joint inflammation did not show this behaviour. It is therefore possible that the maintained regularity index after knee joint injection is, at least in part, due to behavioural compensation. The more persistent impact of ankle inflammation on weight gain is, however, suggestive of increased nociception and might also indicate that some pro-nociceptive effect remains beyond the point in time where gait is normalized. Further studies will be required to test this hypothesis against alternative explanations.

Gait analysis offers a number of possible parameters reflecting limb usage. Temporal aspects include stance-, swing- and step cycle duration and vary with velocity. Different paradigms have been used to quantify changes caused by single joint inflammation, including rotarods where rats are forced to walk [52]–[54], or walkways where rats are observed from underneath and subsequently scored manually or automatically [21], [23], [44], [48], [55]–[59]. Spatial aspects such as paw area, stride length and distance between placement of the two hind paws can be assessed by staining the paws with liquid dye [60], by analysing video recordings [44], [55]–[57] or by using more automated gait analysis systems [19], [20], [23], [58], [59]. In the present work we have focused on parameters that can be translated into weight bearing, as we believe this best reflects the pain and discomfort induced by monoarthritis. Previous studies used mean light intensity per pixel [48], alternatively both light intensity and print area as separate parameters [61]. Our studies with the CatWalk emphasised the importance of both paw contact area, which is markedly reduced by arthritis, and weight load, measured as mean light intensity per pixel [23], and in our opinion, a more accurate estimate of paw usage can be obtained by registering the intensity of all pixels activated through the contact phase, as implemented in the present study.

In the PawPrint paradigm, guarding index is calculated as the difference in percent weight bearing between the hind paws, based on the median value of all steps completed by each paw, and incorporates both the reduced weight bearing of the injected paw and the increased load on the contralateral paw. Using the median prevents undue impact of outliers. We found a slight transfer of weight to the forepaw of the injured side but the effect was less pronounced than previously found in mice where both paw area and light intensity were shifted from the injured paw to all other paws [61]. Several gait parameters are affected by walking speed [62] and the relative distribution of weight between fore- and hind paws may be a confounding factor if forepaw weight bearing is included in the calculation. Normalizing the results of one leg's weight bearing to the percentage of all four legs reduces the impact of individual differences in walking speed, humidity of the paws and body weight. This eliminates the need for procedures to standardise walking speed, which may be difficult to implement as induction of monoarthritis significantly affects this parameter [63]. In principle, a robust measure of walking speed would be a useful complement to weight bearing. As an example, both sedation and analgesia would tend to increase weight bearing on the inflamed joint but would have opposite effects on walking speed. However, analysis of our current data suggests that improvement in algorithm or testing protocol would be required for a reliable implementation of walking speed as a read-out although a tendency to increased walking speed was noted after pharmacological treatment (data not shown).

The second compound parameter calculated by the PawPrint is the regularity index. The program does not correct for incomplete step cycles occurring at the start and end of the analysed distance and for this reason, the parameter rarely reaches 100%. Although the regularity index is reduced in arthritic animals, the measure varies substantially between individuals and is less sensitive than the guarding index. We suggest that guarding index and regularity index together reflect a range of monoarthritis severity, where the first sign is a reduction of weight bearing of the injured paw and more severe affection leads to complete avoidance of stepping on the paw, which then reduces the regularity index.

Recently, considerable attention has been drawn to randomization and blinding issues in animal research [64]–[66]. Inadequate bias control has a major impact on reported effect size in preclinical stroke studies [67] and has been suggested to be a significant problem also in analgesia research [68]–[70]. In the current study, tests are performed by support of a computer program that randomizes treatments between subjects and identifies each rat by a serial number only. The program displays an overview of recorded footprints immediately after each passage and the experimenter can reject the data if there are obvious discrepancies between the observed behaviour and the automated analysis but the group allocation of the animal is not disclosed.

In conclusion, the results show that gait analysis can be an effective method to quantify behavioural effects of single joint inflammation in the rat, useful for pharmacological testing. The window between naïve or control rats and monoarthritic rats is larger during locomotion and there is a more pronounced response to injection of inflammatory agents into the ankle compared to the knee. Ankle injection may therefore be preferable when the objective is to measure changes in weight bearing during locomotion associated with single joint inflammation. Calculating weight bearing during locomotion as introduced here, bringing paw area and light intensity together into one parameter, can be applied to other paradigms as long as paw area and paw pressure is measured. Although the effects of both FCA and carrageenan were concentration related, Freund's incomplete adjuvant was found to be as effective as lower, commonly used, concentrations of FCA and further studies are warranted to address whether higher concentrations have qualitatively different effects on pathophysiology and pharmacology.
